# Machine learning model development to retrospectively predict suicide attempts in the Millenium Cohort Study sample

**DOI:** 10.1192/j.eurpsy.2025.335

**Published:** 2025-08-26

**Authors:** C. Peña Gómez, M. Fradera, M. Caravaca, D. Roche, J. Giraldo, D. Palao

**Affiliations:** 1 Unitat de Neurociència Traslacional, Institut d’Investigació i Innovació Parc Taulí (I3PT-CERCA), Sabadell; 2 Institut de Neurociències (INc), Universitat Autònoma de Barcelona, Bellaterra; 3 Centro de Investigación Biomédica en Red de Salud Mental, Instituto de Salud Carlos III, Madrid; 4 Research Institute for Evaluation and Public Policies (IRAPP), Universitat Internacional de Catalunya (UIC), Barcelona, Spain

## Abstract

**Introduction:**

Suicidal behavior is a complex phenomenon that affects all demographics, with children and adolescents being particularly vulnerable. It is associated with multifactorial conditions that must be considered for the development of more effective prevention strategies. The use of machine learning (ML) models to predict suicide attempts is becoming widespread, as they allow for the simultaneous testing of numerous factors, their complex interactions, and non-linearity in predictive model creation. The Millennium Cohort Study (MCS) is an observational, multidisciplinary cohort study that encompasses a wide range of dimensions, including psychological, genetic, biological, familial, social, and economic factors, as well as traumatic life events, family history, and medical history. This allows for the exploration of their relationship with suicidal behavior throughout individual development using ML models.

**Objectives:**

The aim was to develop a statistical method that applies ML models to retrospectively predict suicide attempts using structured tabular data from an adolescent cohort defined by the MCS.

**Methods:**

The sample consists of 9,824 MCS participants (age 17) who were asked if they had ever purposely hurt themselves in an attempt to end their life. Of these, only 7.4% (725) responded affirmatively. Before starting the modeling phase and fine-tuning any algorithm, several stages were completed: data cleaning, feature extraction and engineering, and feature scaling and selection. We used a wide range of algorithms, from low-complexity (linear regression) to high-complexity (neural networks), while tracking their effectiveness, robustness, generalization, sensitivity, and accuracy.

**Results:**

Even though overall accuracy ranged from 0.83 to 0.87, we generally obtained low f1-scores (˜0.45-0.55) for the targeted class (suicide attempt) and high f1-scores (˜0.95) for the control class. Similar results were observed for precision scores; however, the recall scores were good for both classes, ranging from 0.67 to 0.87. The best performing models were logistic regression and neural networks.

**Conclusions:**

These preliminary results shows that ML models trained with multidimensional data from a young cohort are sensitive in classifying individuals who have attempted suicide. We aim to improve the f1-score and area under the curve (AUC) metrics for the target class through several techniques: over/under-sampling, target encoding, class weight adjustments, ensemble methods, and various neural network architectures.

**Disclosure of Interest:**

None Declared

